# Safety of Five *Tuina* Manipulations in Rats with Deep Vein Thrombosis

**DOI:** 10.1155/2021/6897124

**Published:** 2021-12-06

**Authors:** Ying Zhou, Yumo Zhang, Xiaoyan Zhang, Zhuo Chen, Jian Dong, Chao Yang, Taotao Lv, Tianyuan Yu, Mengqian Lu

**Affiliations:** ^1^School of Acupuncture-Moxibustion and Tuina, Beijing University of Chinese Medicine, Beijing 100029, China; ^2^Department of Traditional Chinese Medicine, Aerospace Center Hospital, College of Aerospace Clinical Medicine of Peking University, Beijing 100049, China; ^3^School of Humanities, Beijing University of Chinese Medicine, Beijing 100029, China; ^4^Dongzhimen Hospital, Beijing University of Chinese Medicine, Beijing 100700, China

## Abstract

**Objective:**

To study the effects of five *tuina* manipulations in rats with deep vein thrombosis (DVT) and to explore how to safely perform *tuina* in the treatment of thrombotic diseases.

**Methods:**

Seventy-two male Sprague-Dawley (SD) rats were randomly divided into the model, pointing manipulation, plucking manipulation, kneading manipulation, pushing manipulation, and pulling manipulation groups (*n* = 12). DVT model was established by incomplete ligation. The *tuina* intervention was started on the next day after modeling and applied once a day 10 times by the manipulation simulators. On the 3^rd^ and 10^th^ days after intervention, respectively, the effects of *tuina* on thrombosis were evaluated based on thrombus elasticity, blood coagulation, fibrinolytic function and blood rheology with the ultrasound elastography, four coagulation tests, enzyme linked immunosorbent assay (ELISA), and hemorheology tests.

**Results:**

In the pointing manipulation group, the strain rate ratio, 6-ketoprostaglandin F1*α* (6-Keto-PGF1*α*), and high shear rate were decreased, and the thromboxane B_2_ (TXB_2_) content was increased (*P* < 0.05). In the plucking manipulation group, the D-dimer and 6-Keto-PGF1*α* contents were increased, prothrombin time (PT) was shortened, and activated partial thromboplastin time (APTT) was activated, and the high shear rate and plasma viscosity were decreased (*P* < 0.05). In the kneading manipulation group, APTT was shortened, and 6-Keto-PGF1*α*, high shear rate, and plasma viscosity were decreased (*P* < 0.05). In the pushing manipulation group, the strain rate ratio, low shear rate, and high shear rate were all decreased (*P* < 0.05). In the pulling manipulation group, both the strain rate ratio and the low shear rate were decreased (*P* < 0.05). The 6-Keto-PGF1*α* changes on the 3^rd^ and 10^th^ days after intervention were opposite in the pushing manipulation group and the pulling manipulation group (*P* < 0.05).

**Conclusion:**

The pointing, pushing, and pulling manipulations seem to be safe in the early period of thrombosis, but the risk is likely to be elevated as the treatment course of intervention increases. The plucking and kneading manipulations potentially have certain risks in the treatment of DVT in rats.

## 1. Introduction

Thrombotic diseases seriously threaten human health and significantly lower the quality of human life globally. They mainly include deep vein thrombosis (DVT), cerebral thrombosis, peripheral arterial thrombosis, pulmonary embolism, myocardial infarction, and a series of other embolic diseases [1]. DVT is a thrombus that forms in the deep veins of the lower extremities or sometimes those of the upper extremities, where blood flow is relatively slow. The clinical manifestations of thrombosis, especially the DVT, are characterized by early onset lower extremity pain, and high-risk individuals often experience dizziness, headache, leg pain, and lower extremity tenderness. The most serious complication of DVT is the dislodgement of the thrombus into the lungs and subsequent pulmonary embolism characterized by high morbidity and mortality. The clinical manifestations of thrombosis are often ignored and misdiagnosed by patients and physicians. The misdiagnosis ratio in European and American countries is as high as 70%, while it is far higher in China [2].

More and more attention has been paid to the study of the formation mechanism and treatment of DVT [3]. DVT is considered to be the result of genetic, environmental, and behavioral activities [4, 5]. The mechanism of thrombosis after venous vascular injury is currently believed to be related to the following factors: abnormal vasoconstriction and spasm, platelet adhesion and aggregation, altered fibrinolytic activity of the vascular intima, and obstacles to the anticoagulation mechanism. When blood vessels are damaged, a series of changes would occur, including increased permeability of capillaries and activated platelets; thus the coagulation process starts, eventually leading to the formation of thrombus [6]. Therefore, the formation of thrombosis is associated with endothelial cells, platelets, function of coagulation and fibrinolytic systems, and hemodynamic abnormalities.

*Tuina* is one of the external treatment methods of traditional Chinese medicine, which has the functions of activating meridians and collaterals, relieving spasm, and activating blood circulation [7, 8]. It can make up for the deficiencies of drug treatment for many diseases and injuries and has an irreplaceable effect. In traditional Chinese medicine, thrombotic diseases are believed to be caused by “blood stasis.” Furthermore, traditional Chinese *tuina* manipulations have good therapeutic effects by activating blood circulation and removing stasis in the treatment of “blood stasis” to prevent the further formation of thrombus. In view of the remarkable curative effect of *tuina* in relieving pain or discomfort, these patients tend to choose *tuina* or other physical therapies [9]. Although *tuina* has been proven to be effective in the treatment of thrombotic diseases, its safety still remains a clinical concern. In recent years, a number of international independent institutions, including the Wolfe-Harris Center for Clinical Studies in the United States, have approved the safety and effectiveness of *tuina* [10] but also alerted physicians to its potential adverse effects and complications [11].

According to the effective, clinical, controllable, and simulable principles, we selected the five most commonly used *tuina* manipulations with the effect of promoting blood circulation and removing blood stasis [1]. The pointing and plucking manipulations belong to the extrusion manipulation, kneading manipulation belongs to the swing manipulation, pushing manipulation belongs to the friction manipulation, and pulling manipulation belongs to the joint movement manipulation. They exert external stresses on the vessel in five ways: local radial force (pointing manipulation), transverse force (plucking manipulation), circular force (kneading manipulation), axial force (pushing manipulation), and distal pulling force (pulling manipulation).

To evaluate the safety of *tuina* on thrombosis, and to provide scientific basis for the safe application of *tuina*, in this paper the safety of five *tuina* manipulations in DVT rats has been evaluated by judging the thrombus elasticity, blood coagulation, fibrinolytic function, and blood rheology.

## 2. Materials and Methods

### 2.1. Animals

Seventy-two clean male Sprague-Dawley (SD) rats (weight 300 ± 10 g) were provided by Sparford Biotechnology Co., Ltd. (SYXK (Jing) 2014-0001), and fed in the barrier environment animal laboratory of Beijing University of Chinese Medicine (SCXK (Jing) 2011-0024). The feeding temperature was 23 ± 2°C, the humidity was 45%, the light and dark period was 12 hours (turning on the light at 8:00 o'clock in the morning), and the rats were provided with food and water freely for one week. After that, the rats were randomly divided into the model group, pointing manipulation group, plucking manipulation group, kneading manipulation group, pushing manipulation group, and pulling manipulation group (*n* = 12). All experimental procedures were approved by the Medical and Experimental Animal Ethics Committee of Beijing University of Chinese Medicine (BUCM-3-20181202-4001).

### 2.2. Modeling Methods

**Figure 1:**
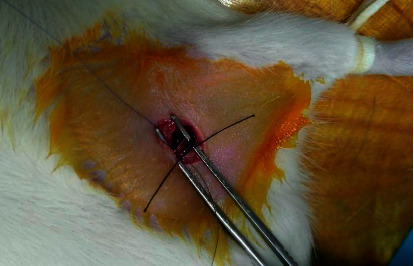
DVT model operation. A 5-0 suture was placed parallel to the vessel; the suture was ligated together with the vessel, and then the suture was withdrawn after ligation.

All rats underwent fasting and water deprivation for 24 hours before modeling. After being anesthetized with intraperitoneal injection of 1% pentobarbital sodium (350 mg/kg body weight), the rats were fixed in the supine position, with the left abdominal-femoral junction being shaved and the uncovered skin sterilized with 10% povidone iodine. Two centimeters longitudinal incision was made along the midpoint of the left inguinal region; thus the left femoral vein was isolated and incompletely ligated around a 5-0 suture at the proximal end to slow down the blood flow (Figure 1). Then the suturing and disinfection were administered layer by layer. The rats were given water without food for 24 hours after surgery, with the wound condition being regularly observed [12, 13].

### 2.3. Intervention Methods

The model group was fed routinely and restrained for 5 minutes daily without *tuina* intervention. In the pointing, plucking, kneading, and pushing manipulation groups, respective manipulations were applied qualitatively on the left femoral vein of rats using the *tuina* manipulation simulator (Patent No. 200710187403.1.), with the stimulation force being 4 N, 60 times per minute, 5 minutes each time. The rats of the pulling manipulation group were placed in the supine position with the forelimbs fixed. The hip joints and knee joints of rats were straightened to 180°, and the ankle joints were dorsally extended to 140° by the pulling manipulation simulator (Patent No. 201620033984.8). Each pulling lasted for 10 seconds and was then suspended for 5 seconds and repeated 20 times. The interventions were started on the next day after modeling, once a day 10 times. On the 3rd and 10th days of intervention, 6 rats of each group were randomly chosen to be sacrificed.

### 2.4. Ultrasound Elastography

**Figure 2:**
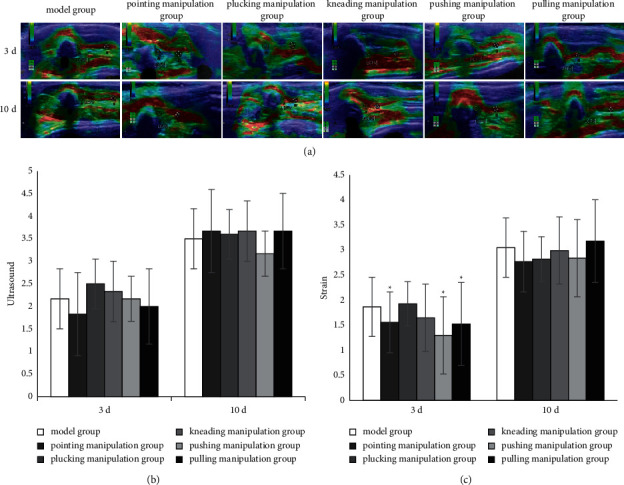
Ultrasonic elastic evaluation. (a) The top and bottom rows show the ultrasound elasticity images on the 3rd and 10th days of intervention, respectively. LCFV: left common femoral vein; A thrombus portion; B peripheral tissue. (b) Ultrasound elasticity score accords with thrombus hardness. The red ROI was rated as 1, red-green was 2, green was 3, blue-green was 4, and blue was 5. (c) The strain rate ratio was calculated based on the difference between the thrombus portion and the perivenous tissue. Data represent mean ± standard deviation (*n* = 6).  ^*∗*^*P* < 0.05 compared with the concurrent model group.

After anesthesia, the body hair around the left femoral vein of rats was debrided. The ultrasound elastic image characteristics of the thrombosis were observed by color Doppler imaging (EUB-7500, Hitachi, Japan) [14]. The region of interest (ROI) was adjusted, and the probe was slid perpendicular to the rats' body surface at a uniform rate; then the image was taken when it was stabilized. The elasticity images were color coded to represent the elasticity of different tissues. As shown in Figure 2(a), green indicates the average hardness of the ROI, red indicates softer than average hardness, and blue indicates harder than average hardness [15]. The thrombus area was selected as zone A and the tissue around veins was selected as zone B. The strain ratio of zone B/zone A was calculated and used as the thrombus hardness value for statistical analysis [16].

### 2.5. Four Coagulation Tests

Rats were anesthetized by 1% pentobarbital sodium and blood was collected from the main abdominal vein and put in sodium citrate vacuum blood collection tube. The blood was centrifuged at 3000 r/min by the high-speed frozen centrifuge (Allegra21R, Beckman, USA) for 10 minutes and then the plasma was taken. The prothrombin time (PT), activated partial thromboplastin time (APTT), thrombin time (TT), and fibrinogen (FIB) of plasma were detected by the automatic coagulation analyzer (Coatron 1800, Beijing Jiuqiang Biotechnology Co., Ltd., China).

### 2.6. Enzyme Linked Immunosorbent Assay (ELISA)

Some blood taken from the main abdominal vein was put in ethylenediaminetetraacetic acid (EDTA) anticoagulation tubes and centrifuged for 10 minutes. Then the plasma was taken and stored at −20°C. The rat D-dimer ELISA kit (CSB-E12984r), rat thromboxane B_2_ (TXB_2_) ELISA kit (CSB-E08047r), and rat 6-keto-prostaglandin F1*α* (6-Keto-PGF1*α*) ELISA kit (CSB-E14411r) were used. All the ELISA kits were bought from *Wuhan Huamei* Bioengineering Co., Ltd. The ELISA kit instructions were strictly followed. The optical density values were read at 450 nm using the enzyme labeler (ELx800, BioTek, USA) and the sample concentrations were calculated for statistical analysis.

### 2.7. Hemorheology Tests

Some blood was put in heparin vacuum blood collection tube and centrifuged for 10 minutes. Then the plasma was taken. The low shear rate, medium shear rate, high shear rate, and plasma viscosity of rats were detected by automatic hemorheology analyzer (LBY-N6 Compact, Beijing Plisson Instruments Co., China).

### 2.8. Statistical Analysis

Data were analyzed by SPSS 25.0 (SPSS Inc., United States), and results were expressed as mean ± standard deviation (*n* = 6). One-way ANOVA and Dunnett test were used when the data followed a normal distribution and homoscedasticity. Nonparametric test was used when the data did not conform to normality or heteroscedasticity. Differences were considered for *P* < 0.05.

## 3. Results

### 3.1. Ultrasonic Elastic Evaluation

On the 3rd and 10th days of intervention, the ultrasonic elastic score showed no significant differences among all the groups (Figure 2(b)). But, on the 3rd day of intervention, the strain rate ratio of the pointing, pushing, and pulling manipulation groups decreased, compared with the model group (*P* < 0.05) (Figure 2(c)).

### 3.2. Coagulation and Fibrinolytic Function

**Figure 3:**
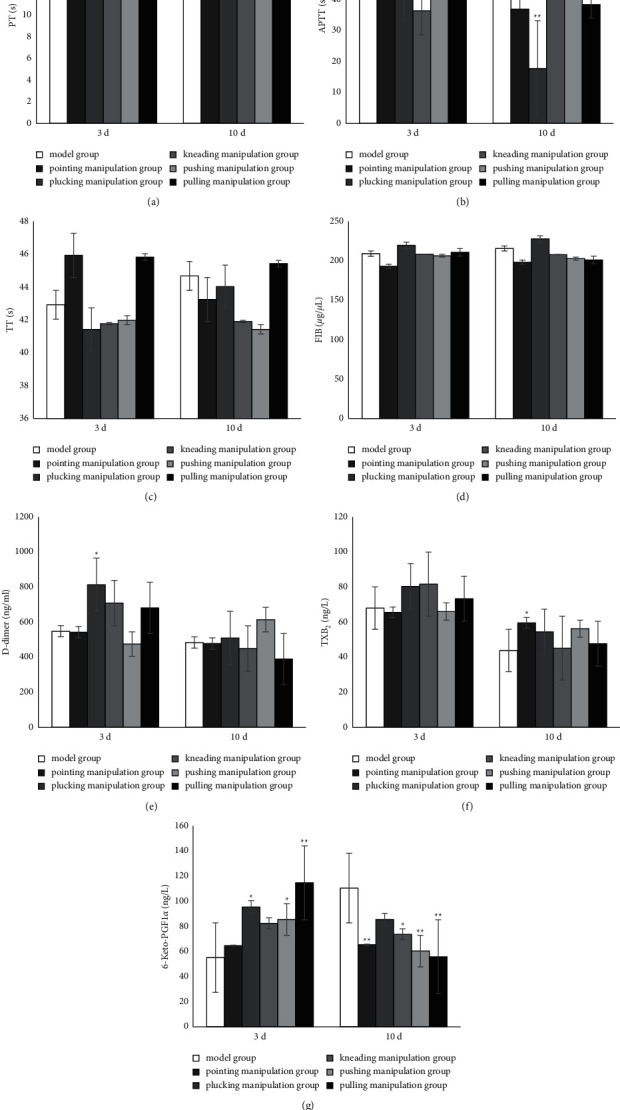
Coagulation and fibrinolytic function. (a) PT, (b) APTT, (c) TT, and (d) FIB were measured using an automatic coagulation analyzer. (e) The expression of D-dimer, (f) TXB2, and (g) 6-Keto-PGF1*α* was detected using ELISA. Data represent mean ± standard deviation (*n* = 6).  ^*∗*^*P* < 0.05,  ^*∗∗*^*P* < 0.01 compared with the concurrent model group.

After 3 days of intervention, PT showed no significant differences among all the groups (Figure 3(a)). After 3 days of intervention, APTT of the kneading manipulation group was significantly shorter than that of the concurrent model group (*P* < 0.05) (Figure 3(b)). After 10 days of intervention, the PT and APTT of the plucking manipulation group were significantly shorter than those of the concurrent model group (*P* < 0.05) (Figures 3(a) and 3(b)). TT and FIB content had no significant differences among all groups (Figures 3(c) and 3(d)).

After 3 days of intervention, the D-dimer content of the plucking manipulation group was higher than that of the model group (*P* < 0.05). But after 10 days of intervention, there were no significant differences among all groups (Figure 3(e)).

After 3 days of intervention, the content of TXB_2_ showed no significant difference among all groups. But after 10 days of intervention, the content of TXB_2_ in the pointing manipulation group showed significant improvement compared with the model group (*P* < 0.05) (Figure 3(f)).

After 3 days of intervention, the 6-Keto-PGF1*α* content of the plucking, pushing, and pulling manipulation groups showed significant improvement compared with the model group (*P* < 0.05). After 10 days of intervention, the 6-Keto-PGF1*α* content of the pointing, kneading, pushing, and pulling manipulation groups was significantly lower than that of the model group (*P* < 0.05), and the content of 6-Keto-PGF1*α* in the plucking manipulation group was lower than that of the model group without significant differences (Figure 3(g)).

### 3.3. Blood Viscosity

**Figure 4:**
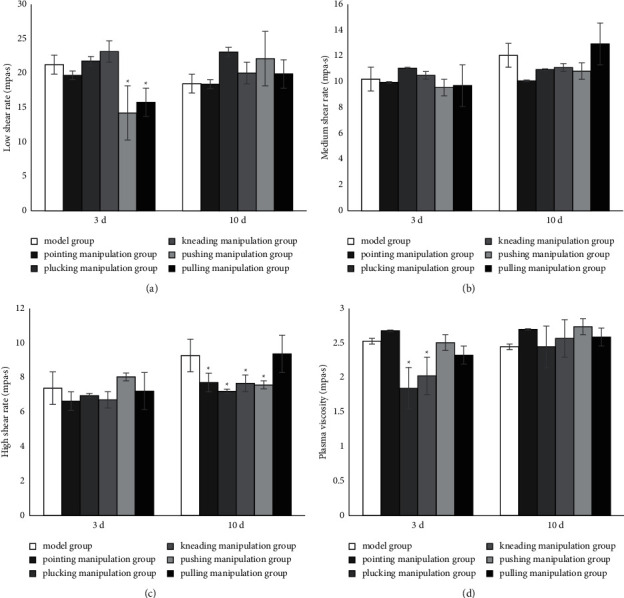
Blood viscosity. (a) Low shear rate, (b) medium shear rate, (c) high shear rate, and (d) plasma viscosity were measured using an automatic hemorheology analyzer. Data represent mean ± standard deviation (*n* = 6).  ^*∗*^*P* < 0.05 compared with the concurrent model group.

After 3 days of intervention, the low shear rate of the pushing and pulling manipulation groups was significantly lower than that of the model group (*P* < 0.05) (Figure 4(a)). The medium shear rate showed no significant differences among all groups (Figure 4(b)). After 3 days of intervention, the high shear rate had no significant differences among all groups. But after 10 days of intervention, the high shear rate of the pointing, plucking, kneading, and pushing manipulation groups decreased, compared with that of the model group (*P* < 0.05). (Figure 4(c)).

After 3 days of intervention, the plasma viscosity of the plucking and kneading manipulation groups was lower than that of the model group (*P* < 0.05). After 10 days of intervention, there was no significant difference among all groups (Figure 4(d)).

## 4. Discussion

In clinic DVT is divided into the acute phase (within 14 days of onset), the subacute phase (15–30 days of onset), and the chronic phase (after 30 days of onset) [17]. At present, there are few animal experimental studies about DVT, and the experimental observation and intervention period are different. Several studies have confirmed [18, 19] that thrombosis of this model begins 24 hours after surgery. Seventy-two hours after modeling, the lumen gets filled with thrombus and there is no adhesion between the thrombus and the lumen. Seven days after modeling, the thrombus contracts and becomes partially adherent to the internal surface of the vein, with no endothelial cells in the area of adhesions, but new capillaries and tissue cells. Fourteen days after modeling, the thrombus is further constricted and adhered to the wall of the vascular wall, and most of the vascular endothelial cells in the adhesion area are lost. This process is generally consistent with clinicopathological development [20, 21]. Therefore, the 3rd and 10th days after intervention were selected as the key time points in our study to investigate the effect of *tuina* on thrombosis.

Changes in thrombus hardness are an important risk factor for thrombus fragmentation and dislodgement. Thrombus hardness is related to the composition of the thrombus and the evaluation of thrombus hardness is a materialistic assessment of the thrombosis risk. Ultrasonic elasticity imaging technology can evaluate thrombus hardness, providing a technical tool for thrombus risk assessment [14]. High elasticity scores and strain rate ratios both indicate high thrombus hardness. Previous studies [22–24] have found that the elasticity of acute thrombosis decreases with time and the difference in elasticity between acute and chronic thrombosis is significant. In this study, the ultrasonic elastography images are predominantly red, red-green, and green, indicating low scores and low thrombus hardness after 3 days of intervention, and green-blue or blue, indicating high scores and high thrombus hardness after 10 days. These differences in ultrasonic elastography can reflect the different stages of DVT to facilitate the prediction of the time of mechanization of DVT [22, 25]. The detection of ultrasound elasticity is both the key to determine the success of the model and the imaging basis for dynamic detection of thrombus formation.

Impaired anticoagulation mechanism is a prerequisite for thrombosis. Current studies have confirmed that the formation of DVT is closely related to the hypercoagulable state of blood [26]. The final common pathway of any coagulation process is the production of thrombin. Therefore, it is important to examine the body's thrombin production to measure the strength of the coagulation system. Prothrombin is evaluated by a combination of the four coagulation tests: PT and APTT mainly reflect the status of the exogenous coagulation system and the endogenous coagulation system, respectively, TT mainly represents the time for conversion of fibrinogen to fibrin, and FIB mainly indicates the fibrinogen content [27]. The shortening of PT and APTT, prolongation of TT, and the increase of FIB are seen in the hypercoagulable state of the blood.

Altered fibrinolytic activity is a direct factor in thrombosis. The fibrinolytic system refers to a process in which the formed fibrin is decomposed and liquefied during blood coagulation. It is a protective physiological response of the body and plays a key role in maintaining vascular permeability and normal blood rheology [28]. The fibrinolytic process can be divided into two phases: activation of fibrinogen and degradation of fibrin [29]. When a fibrin clot is formed, in the presence of plasminogen activator (t-PA), plasminogen is activated and converted to plasmin, and plasmin degrades the fibrin clot into various soluble fragments to form the fibrin degradation products (FDP) [30, 31]. D-dimer is a specific marker of the fibrinolytic process and is one of the sensitive indicators of hypercoagulable and hyperfibrinolytic state of DVT. If the secondary fibrinolytic condition happens, D-dimer in the plasma would increase rapidly, so it is a major marker for determining thrombosis in clinic [32, 33].

Platelets play an important role in thrombosis. When the vascular endothelium is damaged or blood flow is slow, the platelet activation process is initiated, followed by platelet adhesion, aggregation, and release reactions, and the contents hidden in the vascular granules and metabolites from the activation process are released. Thromboxane A_2_ (TXA_2_) and Prostaglandin-I-2 (PGI_2_) are endogenous substances that have been identified as the most potent regulators of platelet function. PGI_2_ acts as a vasodilator and inhibits platelet aggregation, while TXA_2_ exerts its potent platelet aggregation and vasoconstriction functions [34]. Because the half-life of them is only a few minutes, during which time TXA_2_ is quickly hydrolyzed to the inactive TXB_2,_ while PGI_2_ is rapidly oxidized to 6-Keto-PGF1*α*, they are difficult to measure directly. Therefore, the stable metabolites of plasma TXA_2_ and PGI_2_ (i.e., TXB_2_ and 6-Keto-PGF1*α*) are generally measured to represent their levels. Recent studies have shown [35, 36] that TXA_2_/PGI_2_ imbalance is closely associated with the development of DVT and the levels of TXB_2_ and 6-Keto-PGF1*α* are closely related to DVT formation with high sensitivity.

Hemodynamic abnormality is a major factor in thrombosis. The three main factors affecting whole blood viscosity are erythrocyte aggregation, deformability, and plasma viscosity. The measurement of blood rheology is a good indicator for the assessment of thrombosis, and blood viscosity at different shear rates has different rheological meanings. The apparent viscosity at high shear rate mainly reflects erythrocyte deformation, when there is generally no aggregation. The apparent viscosity at medium shear rate reflects the viscosity of erythrocytes when they are both deformed and not aggregated. The apparent viscosity at low shear rate reflects the viscosity of blood flow under conditions of erythrocyte aggregation, when there is no distortion. It has been suggested that blood viscosity is significantly elevated in patients with thrombophilia compared to normal subjects [37].

The results of pointing manipulation group showed that, after 3 days of intervention, the strain rate ratio decreased and there were no significant differences in the other 12 indicators, suggesting that pointing manipulation does not increase the formation of DVT. After 10 days of intervention, the expression of TXB_2_ increased and 6-Keto-PGF1*α* decreased, with no significant differences in the other 11 indicators. Therefore, pointing manipulation is probably safe for early interventions after molding, but a risk of exacerbating thrombosis is likely to exist as the intervention days increase.

The results of the plucking manipulation group showed that, after 3 days of intervention, the expression of D-dimer and 6-Keto-PGF1*α* increased and plasma viscosity decreased, with no significant differences in the other 10 indicators. After 10 days of intervention, PT and APTT shortened, 6-Keto-PGF1*α* and high shear rate decreased, and there were no significant differences in the other 9 indicators, suggesting that the plucking manipulation has certain risk of aggravating DVT.

The results of the kneading manipulation group showed a decrease in APTT and plasma viscosity after 3 days of intervention, with no significant differences in the other 11 indicators. After 10 days of intervention, the expression of 6-Keto-PGF1*α* and high shear rate decreased, with no significant differences in the other 11 indicators. Even though the APTT result of 10 days had no difference, other indicators changed. It is suggested that thrombosis is affected by many factors, at different stages, different manipulations may affect its different aspects, and the kneading manipulation has slight risk of aggravating DVT in 3 days and 10 days.

The results of the pushing manipulation group showed that, after 3 days of intervention, the low shear rate and strain rate ratio decreased and 6-Keto-PGF1*α* increased, while there were no significant differences in the other 10 indicators, suggesting that the pushing manipulation has the potential to reduce DVT safely. After 10 days of intervention, the expression of 6-Keto-PGF1*α* and high strain rate decreased, with no significant differences in the other 11 indicators. This suggests that the pushing manipulation seems to be safe in the early stage, but there might be certain risks of aggravating thrombosis as the intervention days increase.

The results of the pulling manipulation group showed a decrease in strain rate ratio and low shear rate, and an increase in 6-Keto-PGF1*α* after 3 days of intervention, with no significant differences in the other 10 indicators. This suggests that the pulling manipulation could reduce DVT with high safety. After 10 days of intervention with the pulling manipulation, the expression of 6-Keto-PGF1*α* decreased and there were no significant differences in the other 12 indicators. This suggests that the pulling manipulation is likely to be safe in the early postmodeling period, but there might be a risk of increased thrombosis as the intervention days increase.

At present, there are few related basic researches on the effects of *tuina* manipulations on thrombotic diseases. The previous research mostly focused on the influence of manipulation on the anatomical structure of the neck and related biomechanics. Some studies have confirmed that the cervical rotatory manipulation affected the hemodynamic indicators of vertebral artery and basilar artery, reduced the tensile properties of blood vessels, and increased the risk of plaque shedding [38–42]. But they also found that the cervical rotatory manipulation had no obvious effect on the ultrastructure of the vascular intima, and it could be safely applied to patients with early atherosclerosis [42].

Our research showed that the choice of different *tuina* manipulations and the intervention timing are important for the safe treatment of DVT, because of the different ways of applying force of the five manipulations. The plucking and kneading manipulations both showed certain risks after 3 days of intervention, indicating that the manipulations which have strong force and penetration may increase the risk of thrombosis. The pointing, pushing, and pulling manipulations were safe after 3 days of intervention. This may be because the manipulations that are less powerful and mainly apply force in the superficial layer of the skin are safer in the early stage. Therefore, the *tuina* therapist should be careful to identify whether the patient has a thrombotic disease before treatment and choose the appropriate manipulation and treatment modality according to the patient's age and physique. After 10 days of intervention, it was shown that all the manipulations were dangerous to some degree when applied on DVT rats. The 10-day period exists as a critically dangerous time for the intervention with the five manipulations, as the DVT may be in the acute phase with many unstable factors. Because of the instability of the DVT process, this study suggests that the best period for *tuina* intervention is in the early stage of DVT or when the DVT has not formed.

This study has some limitations. Firstly, although the DVT model of this study is successful, the femoral vein of rats is relatively thin and it is slightly difficult to operate. Therefore, large animal model, such as rabbits or monkeys, can better simulate the conditions of human beings. Secondly, now the studies about the exact formation time of DVT and the different periods of thrombosis are still at a preliminary stage. The optimal timing of *tuina* on DVT needs to be further investigated after the specific stage of DVT in animal models is more clearly defined. Thirdly, in the current experimental design, the variables of all manipulations are single. We should continue to study the effect of different strengths or frequencies of *tuina* manipulations on DVT.

## 5. Conclusions

In conclusion, from the perspective of ultrasound elasticity, there seems to be little risk regarding the five *tuina* manipulations in DVT rats. Judging from the functions of the coagulation and fibrinolytic systems, the pointing, pushing, and pulling manipulations are likely to be safe, while the plucking and kneading manipulations have certain risks in DVT rats. However, as the intervention days increase, the pointing, pushing, and pulling manipulations also show certain risks. In terms of blood viscosity, little risk could be observed regarding the five *tuina* manipulations in DVT rats. The intervention timing and strength of the five manipulations in DVT rats need to be further studied. It is recommended that when we treat patients with a thrombotic disease in clinic, we should use gentle manipulations, such as pointing and pushing manipulations and use plucking and kneading manipulations with caution.

## Data Availability

The data used to support the findings of this study are available from the corresponding author upon request.
